# HLA-C and HIV-1: friends or foes?

**DOI:** 10.1186/1742-4690-9-39

**Published:** 2012-05-09

**Authors:** Donato Zipeto, Alberto Beretta

**Affiliations:** 1 Laboratory of Molecular Virology, Department of Life and Reproduction Sciences, Section of Biology and Genetics, University of Verona, Strada le Grazie 8, 37134, Verona, Italy; 2 Infectious Diseases Department, IRCCS Ospedale San Raffaele, Via Stamira d’Ancona 20, 20127, Milan, Italy

**Keywords:** MHC class I, HLA-C, Open conformers, HIV-1, CTL, NK cells, KIR, β_2−_microglobulin

## Abstract

The major histocompatibility complex class I protein HLA-C plays a crucial role as a molecule capable of sending inhibitory signals to both natural killer (NK) cells and cytotoxic T lymphocytes (CTL) via binding to killer cell Ig-like receptors (KIR). Recently HLA-C has been recognized as a key molecule in the immune control of HIV-1. Expression of HLA-C is modulated by a microRNA binding site. HLA-C alleles that bear substitutions in the microRNA binding site are more expressed at the cell surface and associated with the control of HIV-1 viral load, suggesting a role of HLA-C in the presentation of antigenic peptides to CTLs. This review highlights the role of HLA-C in association with HIV-1 viral load, but also addresses the contradiction of the association between high cell surface expression of an inhibitory molecule and strong cell-mediated immunity. To explore additional mechanisms of control of HIV-1 replication by HLA-C, we address specific features of the molecule, like its tendency to be expressed as open conformer upon cell activation, which endows it with a unique capacity to associate with other cell surface molecules as well as with HIV-1 proteins.

## Review

### Introduction

The major histocompatibility complex (MHC) class I loci are the most polymorphic mammalian genes. The human MHC class I system includes 3 main loci, namely HLA-A, -B and -C, and some non-classical HLA genes such as –E, -F and –G. The HLA-B locus is the most polymorphic, with 1795 proteins known, followed by HLA-A with 1290, and HLA-C with 946 (http://www.ebi.ac.uk/imgt/hla/stats.html). Among the classical MHC class I genes, HLA-C has the least allelic variability [1].

MHC class I molecules consist of a 45 kDa heavy, α-chain (divided in 3 subdomains, α1, α2 and α3) non-covalently associated with a light β chain, the β_2_-microglobulin (β_2_m), and a short (8-11mer) peptide antigen derived from the degradation of intracellular proteins [2]. MHC class I assembly occurs in the lumen of the endoplasmic reticulum (ER) with the help of molecular chaperons like calreticulin, ERp57 and tapasin [3]. After leaving the ER the trimeric complexes egress through the Golgi complex to the plasma membrane. At the plasma membrane they can interact with two main types of ligands: the T cell receptor (TCR) of CD8+ T cells and natural killer cells (NK) receptors including immunoglobulin receptors (KIRs), leukocyte immunoglobulin receptors (LILRs) [4,5] and NKG2A/C [6].

It is generally agreed that HLA-C is surface-expressed at levels much lower (sometimes said to be ten-fold lower) than HLA-A and HLA-B [7] despite similar levels of transcription and translation [8]. Several authors provided evidence for transcriptional, post-transcriptional, as well as post-translational impairments (reviewed in [9]). Due to such considerable redundancy in the mechanisms accounting for low HLA-C expression, different HLA-C alleles may be affected to various degrees in different cell types or biological conditions. A post-translational biosynthetic bottleneck appears nevertheless to be common, in that it causes the backward accumulation of chaperone-associated and deeply unfolded HLA-C heavy chain conformers of all the 8 serologically defined (Cw*01 through Cw*08) alleles. This bottleneck is due to the HLA-C-specific KYRV heavy chain motif, initially noted by Zemmour and Parham [10] and subsequently proposed to provide HLA-C with reduced binding groove plasticity and selective peptide-assembly properties [9].

The mature trimeric HLA-C complexes expressed at the cell surface tend to dissociate and generate a pool of heavy chains, lacking peptide and β_2_m. The surface expression of free HLA-C heavy chains, also called “open conformers”, is a hallmark of activated and transformed cells, including lymphocytes [11,12]. The expression of HLA-C open conformers is also upregulated by treatment with IFN-γ in human trophoblasts [13] and in other human cells belonging to a variety of lineages [12].

An additional selective feature of HLA-C lies in its cytoplasmic domain containing a di-hydrophobic internalization signal (DXSLI), which is specifically inhibited upon macrophage differentiation, allowing high HLA-C surface expression on macrophages [14]. The specific induction of HLA-C expression on differentiated macrophages may play a role in the down-modulation of the normal CD8+ T cell response to specifically limit the lysis of antigen-presenting cells (APC) that cross-present the antigen [14]. Thus, it appears that complex mechanisms tightly regulate the expression of HLA-C, maintaining significant intracellular levels and limiting surface expression on resting cells, but also rapidly mobilizing the intracellular pool to allow surface expression after cell activation or differentiation.

HLA-C presents antigen to cytotoxic T lymphocytes less efficiently than either HLA-A or -B [15]. In contrast, HLA-C is an extremely good ligand for KIR receptors on NK cells and protects target cells from lysis mediated by NK cells [16]. The HLA-C-mediated protection is relevant to many viral infections, including HCV [17,18] and HIV-1 [19-24]. Inhibitory HLA-C/KIR interactions influence the outcome of HCV infection and confer protection [17]. Several lines of evidence highlight the importance of NK cells in the setting of HIV-1 infection [19-24]. Furthermore, HIV-1 has developed a sophisticated pathway to modulate, to its own advantage, the surface expression of MHC class I molecules. The suppression of HLA-A and -B, but not -C, results in escape of immune recognition of HLA-A and –B peptides by CTL. At the same time an efficient NK inhibitory signal at the surface is maintained [25-31], pointing to a selective advantage for HIV-1, which results in maintenance of a sufficient level of cell-surface HLA-C expression.

Further complexity has been added by the discovery of a post-transcriptional mechanism of HLA-C expression control [32]. Specifically, such control depends on the expression of an intact microRNA binding site in the 3′ UTR of the HLA-C gene. Strong genetic association between HLA-C alleles with a mutated microRNA binding site, higher protein expression, and control of HIV-1 replication was reported. These findings are in contradiction with the view of HLA-C as an inhibitory molecule and call for a general reappraisal of the role of HLA-C in HIV-1 infection.

### MHC class I genes and control of HIV-1

Experimental evidence shows that CD8+ T cells play a central role in controlling HIV-1 viremia during primary infection as well as in the long-term suppression of viral replication [33]. The correlation between expression of specific MHC class I alleles and HIV-1 control strongly supports this idea. Early studies performed in the late 1980′s on small cohorts of AIDS patients reported an increased frequency of C*04 and B*35 in HIV-1-infected patients compared to non-infected controls [34], an elevated C*04 frequency in patients with Kaposi’s sarcoma and of C*07 in patients with opportunistic infections [35]. Subsequent studies reported an increased prevalence of C*07 in HIV-1 positive individuals [36]. A larger study published in 1999 established the association of C*04 and B*35 with rapid development of AIDS-defining conditions in Caucasians [37], and also showed that maximum homozygosity at the HLA-B locus was more detrimental than homozygosity at either HLA-A or -C. A follow up study from the same group showed that peptide presentation properties of particular B*35 subtypes explain much of this association [38]. The dominance of HLA-B in the control of HIV-1 infection is supported by several studies which associated HLA-B*57, B*5801 and B*27 with slower disease progression rates (time to AIDS) and strong virologic (viral load) and immunologic control (CD4 count) [39-48], while B*35, B*5802 and B*18 were associated with ineffective control of viral replication and rapid progression to AIDS [37,43,44,47,49,50]. In addition, a significantly greater number of CD8+ T cell responses are HLA-B restricted, compared to HLA-A and -C. Variation in viral set-point, absolute CD4 count, and rate of disease progression are strongly associated with particular HLA-B, but not –A or –C alleles [43]. Recently, a study on blood donors in China who were contaminated by a narrow source blood-born virus showed that HLA-B exerts a greater selection pressure on HIV evolution than other HLA molecules [51].

On the other hand, there is evidence of interdependent protective effects of the HLA-C*0401-B*8101, HLA-C*1203-B*3910 and HLA-A*7401-B*5703 haplotypes that cannot be explained solely by linkage to a protective HLA-B allele. Therefore, although individual HLA alleles, particularly HLA-B, can have a strong impact, HIV-1 control is likely to be influenced by the additive effect of some or all the other HLA alleles present [45]. Other HLA alleles combinations like HLA-B*5701-C*0602, HLA-B*2705-C*0102, or HLA-B*3801-C*1203 also exert a strong effect on non-progression, reinforcing the hypothesis of an additive effect of protective HLA alleles [48,52]. More recently, a study on HIV-infected children identified HLA-C*02 as a protective allele while confirming a strong protective effect of the B*27 allele [53].

HLA-C drives viral evolution through the selection of CTL escape mutants. A recent study used three phylogenetic correction methods across a full HIV-1 subtype C proteome and identified amino acids conferring either susceptibility or resistance to CTLs [54]. Three hundred and ten groups of HLA-amino acid associations were identified, each predicted to be related to single epitope/HLA combinations (immunological set). The immunological set with the most individual associations was found within Nef and included a B*44-restricted epitope (molecules of the Bw4 subfamily of HLA-B alleles). The next largest immunological sets were in Nef, Tat and Pol and included six associations involving a C*0404-restricted epitope as well as two B*42-restricted epitopes [55]. CTL escape mutants were also identified in a Cw*12 restricted pol epitope [54].

Recently, genome-wide association studies (GWAS) were applied to the study of genetic control of HIV-1. These studies do not rely on candidate gene selection and have the potential to identify new genomic regions and pathways affecting human diseases [56]. The first study identified genetic polymorphisms associated with viral load during the asymptomatic phase of infection, as well as time to HIV-1 disease progression [57]. Two independently acting groups of polymorphisms associated with HLA loci B and C were identified explaining 9.6% and 6.5%, respectively, of the total variation in viral set point. The first polymorphism, located in the HLA complex P5 (HCP5), is in high linkage disequilibrium with the B*5701 allele [58]. The second polymorphism (C/T) is located in the 5′ region of the HLA-C gene 35 kb away from transcription initiation. This single nucleotide polymorphism (SNP) associates with differences in HLA-C expression levels. The protective allele (C SNP) leads to lower viral load and is associated with higher expression of HLA-C [57].

A second GWAS (The International HIV Controllers Study) [59] conducted in a multiethnic cohort of HIV-1 controllers and progressors analyzed the effects of individual amino acids within the classical HLA proteins. In the group of individuals of European ancestry, 4 independent markers of association were identified, which included the −35 kb and the HCP5 SNPs previously identified [57]. In addition, B*57.01, B*27:05, B*14/C*08:02, B*52, and A*25 were identified as protective alleles and B*35 and C*07 as risk alleles. The study also evaluated the impact of specific amino acid positions within the HLA molecules and identified six residues as independent markers associated with control of HIV-1: Arg^97^, Cys^67^, Gly^62^, and Glu^63^, all in HLA-B; Ser^77^ in HLA-A and Met^304^ in HLA-C. Collectively these residues explained 20% of the variance. Interestingly, all these residues, with the exception of Met^304^ of HLA-C, are in the peptide-binding groove. Position 304 is a biallelic variant (Val/Met) located in the transmembrane domain of HLA-C, which is in moderate linkage disequilibrium with the −35 SNP associated with HLA-C expression level. Therefore mechanisms other than peptide selection by polymorphic HLA-C residues may underlie the association between HLA-C expression and HIV-1 control [59]. Collectively, the data generated by the GWAS confirmed the dominance of HLA-B in host control of HIV-1, but also revealed a novel mechanism of control related to HLA-C expression.

The association between the −35 SNP and HIV-1 viral load has been questioned in a work by Corrah and colleagues who showed that differences in HLA-C expression across −35 SNP genotypes can be attributed primarily to the very low expression of a single allelic product, HLA-Cw*07 [60].

Some individuals with the reported protective -35CC SNP genotype exhibit high viral loads. HIV-1 variants isolated in these patients counteract HLA-C-mediated immune control of HIV-1 by enhancing Nef mediated viral infectivity. This was not due to an acquired ability of Nef to down-modulate HLA-C or in a more efficient ability to down-modulate HLA-A and –B, but rather to an indirect mechanism interfering with T-cells function and MHC-II antigen presentation [61].

### Post-transcriptional regulation of HLA-C expression

Recently, Kulkarni et al. [32] discovered that the −35 kb SNP is not the causal variant for differential HLA-C expression, but rather marks another polymorphism in the 3′ UTR region of HLA-C that directly controls the levels of HLA-C [32]. The 3′ UTR variant affects binding of a microRNA (miR-148a) to its target site resulting in relatively low surface expression of HLA-C alleles that bind this miRNA (“inhibited alleles”) and high expression of HLA-C alleles that escape the miRNA control (“escape alleles”). HLA-C alleles, such as C*01, C*03, C*04, C07*, C*14 and C*17, contain an intact miR-148 binding site, and their expression is down-regulated (“inhibited alleles”). On the contrary, C*02, C*05, C*06, C*08, C*12, C*15 and C*16, containing a disrupted binding site, are highly expressed (escape alleles). These findings offer a rational explanation of different HLA-C expression levels initially reported to be associated with the −35 kb SNP and are also consistent with previous genetic association studies which identified C*04 and C*07 (inhibited alleles) as risk alleles [34-36] and C*02 and C*12 [52] (escape alleles) as protective alleles [53]. A subsequent study showed that the common ancestor of all extant HLA-C alleles was suppressed by miR-148a and that substitutions that prevent miR-148a binding arose by a recombination event between an ancestral HLA-C allele and an HLA-B allele of the HLA-B*07-like lineage. This resulted in conversion of the 3′ UTR of the original HLA-C allele by the paralogous HLA-B region. This event is likely to have occurred 3 to 5 million years ago, after the split of humans from great apes, resulting in HLA-C variants that escape control by miR-148 [62]. The frequency of the escape alleles in worldwide populations is high, constituting an estimated 32.8% of HLA-C alleles. In addition, many polymorphisms and functional motifs specific for HLA-C alleles are present in both escape and inhibited lineages indicating that selection has been instrumental in the diversification of the escape lineage [62].

On the other hand, the inhibited alleles may also provide benefit, since they have been maintained in evolution, and they have been identified as restricting elements for HIV-specific CTL clones [63-68]. Notably, with the exception of one CTL clone restricted to HLA-C*15 (an escape allele) [63], all other clones are restricted to inhibited HLA-C alleles like HLA-C*01 [69], -C*03 [63,66], -C*04 [64,65,67,68] and -C*07 [63]. Furthermore, Cw*07 restricted CTLs were detected in non-progressor patients [70], and a Cw*04 restricted epitope was associated with the generation of CTLs escape mutants [55].

### HLA-C and NK cells

NK cells express receptors (KIR) which interact with the α1 domain of MHC class I molecules resulting in inhibition or activation of cytolysis [71]. HLA-C alleles are the main inhibitory ligands for KIRs, protecting target cells from lysis mediated by NK cells [16,72]. HLA-C/KIR interactions have been shown to play a key role in placentation [73].

KIR distinguishes HLA-C alleles into two groups, namely C1 and C2, depending on a dimorphism present in the α1 domain [74,75]. In particular, KIR2DL2 and 3 bind with greater affinity to HLA-C group 1, while KIR2DL1 bind to HLA-C group 2 [76]. Homozygosity for HLA-C1 alleles and KIR2DL3 is associated with resolution of HCV infection as compared to homozygosity or heterozygosity for HLA-C2 [17]. In the case of HIV-1, resistance to infection has been associated with the presence of inhibitory KIR in the absence of their HLA-C ligands [20] The activating receptor KIR2DS4 was associated with high viral load and accelerated transmission although this effect was independent of the putative ligand HLA-Cw*04 [22]. HLA-C1 alleles were also associated with an increased magnitude of NK cell response in HIV-1 infected subjects [24]. The KIR2DL2-HLA-C1 compound was also shown to drive viral evolution *in vivo* since KIR2DL2-associated HIV-1 sequence polymorphisms were shown to enhance the binding of inhibitory KIRs to HIV-1 infected CD4+ T cells and to reduce anti-viral activity of KIR-positive NK cells thereby enabling HIV-1 to escape the potential protective role of KIR [77].

Another KIR/HLA compound genotype relevant to HIV-1 control is KIR3DL1 and KIR3DS1, which encode receptors for molecules of the Bw4 subfamily of HLA-B alleles. The activating allele, KIR3DS1, when present in combination with Bw4, is associated with lower viral load, slower decline of CD4+ T cells and delayed progression to AIDS [21]. KIR3DS1 is also associated with strong inhibition of viral replication [19]. The importance of the KIR3DL1/KIR3DS1 locus in control of viral set point was recently confirmed by a GWAS that assessed the copy number variant of KIR3DL1/KIR3DS1. The study showed that an increase in KIR3DS1 count associates with a lower viral set point if its putative ligand is present, as does an increase in KIR3DL1 count in the presence of KIR3DS1 and the appropriate ligands for both receptors, suggesting that the relative amounts of activator and inhibitory KIR regulate the expansion of antiviral NK cells [23].

It should be considered, however, that inhibition by HLA-B allotypes is less common compared to inhibition by HLA-C since it has only been shown for the Bw4 subfamily. In contrast, since nearly all KIR allotypes worldwide contain KIR2DL1 along with either KIR2DL3 or KIR2DL2, HLA-C molecules almost always inhibit a subset of each individual’s NK cell population.

Although generally considered NK cell receptors, KIR are also expressed by a large fraction of effector memory T cells, which, like NK cells, are immediate effector cells that are cytotoxic and produce IFN-γ (reviewed in van Bergen and Koning [78]). On cytotoxic T cells, KIRs modulate signals driven by the T-cell receptor and inhibit cytokine secretion, degranulation, and proliferation. In HIV-1 infection, KIR expression on cytotoxic T cells is progressively upregulated, and this correlates with the level of viral replication [79]. Interestingly, the upregulation of KIR occurs in individuals who do or do not express the respective KIR ligands, suggesting a possible ligand-independent blockade of TCR activation [79].

### Virion HLA-C molecules and HIV-1 infectivity

During the process of budding from the cell membrane, MHC class I and II molecules are incorporated into the HIV-1 envelope together with other cell proteins [80-85]. HIV-1 viral particles have been shown to carry more MHC molecules than Env trimers [86-88]. The process of host cell protein incorporation is neither random nor dependent on the amount of protein on the cell membrane, since some highly expressed proteins such as CD4, CD45, CCR3, CCR5 or CXCR4, are not incorporated. The preferential incorporation in the budding envelopes suggests a role in the pathogenesis of HIV-1 [80]. For instance, virion-associated MHC class II molecules have been shown to confer higher viral infectivity, possibly enhancing CD4 binding [89,90].

There is evidence that virion HLA-C molecules play a role in HIV-1 infectivity. Fusion between the viral envelope and the cell membrane is increased by HLA-C [91]. This effect is not due to binding to a specific cellular ligand, since the natural CD8 MHC class I ligand is not expressed on cells susceptible to HIV-1 infection. Cosma et al. reported that MHC class I negative cells are non-permissive for replication of primary HIV-1 isolates, and transfection of HLA-C restores their permissivity. This effect of HLA-C is also evident, but to a lesser extent, with T cells line-adapted viruses [92]. HLA-C molecules associate non-covalently with the HIV-1 envelope glycoprotein gp120 [92]. Gp120 purified from HLA-C positive virions displays conformational changes, which include an enhanced exposition of epitopes normally available upon CD4 binding. In addition, virus incorporation of HLA-C reduces the susceptibility to neutralizing antibodies [92].

The effect of HLA-C on HIV-1 infectivity was further studied by Matucci et al. [93] who showed that fusion efficiency is reduced in HLA-C silenced cells and that pseudoviruses produced in HLA-C silenced cells are significantly less infectious compared to those produced in non-silenced cells. The study also confirmed the association between HLA-C and gp120 demonstrating that HLA-C molecules are detectable, together with gp120, CD4 and CCR5 within molecular complexes which form on cells during the process of HIV-induced cell-to-cell fusion (fusion complexes).

These studies addressed the association between gp120 and HLA-C using an HLA-C specific monoclonal antibody, L31 [11], which was originally derived from a mouse immunized with purified HIV-1 virions [94] and later shown to bind all HLA-C and a few rare HLA-B open conformers [94,95]. L31 binding to HLA-C is strictly dependent on the presence of aromatic amino acids (Phe or Tyr, present in HLA-C and certain HLA-B alleles) at position 67 of the locus-specific motif on the α1 helix [11], which is buried deep in the binding groove [96]. The gp120/HLA-C association appears, therefore, to selectively involve the open conformers of HLA-C. Enhancement of HIV-1 infectivity was observed in cell lines transfected with three inhibited HLA-C alleles (C*03, *04 and *07) and one escape allele (C*12) expressed in heterozygosity with C*07 (see Table 1).

**Table 1:** principal characteristics of the main HLA-C variants

**HLA-C allele**	**miR418a site/expression**[32,34-36,52,53,59]	**−35 kb SNP**[32]	**Val/Met 304**[57,59]	**KIR group type**[74,75]	**CTL restriction**	**Association with control**	**Association with progression**	**Increase of HIV-1 infectivity**[92,93]
01.02	Intact/Low	C	Val*	C1	[69]			NT
02.02	Deleted/High	C	Val*	C2		[53]		NT
03.03, .04	Intact/Low	T	Val*	C1	[63,66]			Yes
04.01	Intact/Low	T	Val*	C2	[63-68]		[34,37]	Yes
05.01	Deleted/High	C	Met*/Val*	C2				NT
06.02	Deleted/High	C	Met*/Val*	C2				NT
07.01, .02, .04	Intact/Low	T	Val	C1	[63,70]		[36]	Yes
08.01, .02, .04	Deleted/High	C	Met*/Val*	C1				NT
12.02, .03	Deleted/High	C	Met*/Val*	C1*	[54]	[54]		Yes ?**
14.02	Intact/Low	C	Val*	C1*				NT
15.02, .05, .06	Deleted/High	T	Met*/Val*	C2*	[63]			NT
16.01, .02, .04	Deleted/High	T	Val*	C1*				NT
17.01	Intact/Low	T	Val*	C2*				NT

*: data obtained by sequence alignment analysis.

**: C*12/C*07.

*NT* not tested.

In T lymphocytes, HIV-1 assembly, budding, and release occur at the plasma membrane, while in macrophages they occur in intracellular compartments such as the late endosomes [97]. This process is coordinated by the viral protein Gag [98]. MHC class II molecules, which are expressed on macrophages and activated T cells, relocate Gag to late endosomes and substantially decrease viral production and release from the cell surface [97]. HLA-C molecules, like other MHC class I molecules, are mobilized from late endosomes to the cell surface [99] and may therefore play a similar role, acting as chaperones of the viral envelope proteins and participating to the process of virus assembly and budding.

MHC class I open conformers have been shown to be able to cis-associate both with themselves and with a variety of membrane receptors, including CD3, CD8αβ, CD25, Ly49A and IL-15Rα [100] and tend to maintain an ordered and non-denatured structure. HLA-C binds weakly to β_2_m and has the longest reported half-life compared to other free MHC class I heavy chains [101]. This property may confer to HLA-C open conformers a selective propensity for associating with viral proteins.

## Conclusions

HLA-C can influence the outcome of HIV-1 infection in at least three distinct and potentially opposite ways: (a) promoting effective CTL recognition and lysis of HIV-infected cells, (b) inhibiting NK cell recognition and lysis of infected cells, and (c) favoring the formation of infective virions via its association with the envelope protein. The extent to which genetic polymorphisms associated with control of HLA-C expression levels influence these three properties of the molecule is largely unknown. The strong genetic association between high expression of HLA-C and control of HIV-1 suggests that, at least, in the initial setting of HIV-1 infection, the role of HLA-C in inducing an effective CTL response dominates over other functions of the molecule. However, the role of the escape (highly expressed) HLA-C allotypes in the induction of CTL responses needs further investigation, since only a handful of HLA-C specific CTL clones restricted to escape alleles have been reported so far, while the majority of clones are restricted to inhibited alleles [102] (see Table 1). In addition, no significant associations between HIV-1 control and amino acid positions located in the HLA-C peptide binding pocket were identified by the GWAS, as it would be predicted if selection of antigenic peptides by HLA-C were to play a major role in control [59], by analogy with HLA-B. Thus, if high cell surface expression of HLA-C suggests a more potent induction of cytotoxic T cell responses, other properties and functions of the escape alleles may contribute to the control of viral replication. The main feature of HLA-C molecules is their poor assembly efficiency, which results in intracellular accumulation as open conformers. Differences in binding affinity to their cognate ligand, β_2_m, or to alternative ligands may confer to each allelic variant of HLA-C a selective capacity to function as accessory molecule in the process of virus assembly and budding. In addition, preferential association with alternative ligands may affect the pattern of cell surface expression of the alternative isoforms. Accordingly, escape alleles may have a higher affinity with β_2_m, resulting in higher surface expression of trimeric complexes, but also lower propensity to associate with viral proteins. This property would confer to lymphoid cells of individuals bearing HLA-C escape allotypes a higher sensitivity to CTLs, but also an intrinsically lower capacity to support viral replication. Studies addressing the characterization of the HLA-C molecular isoforms on infected cells expressing escape versus deleted alleles are warranted to gain novel insights into the potential protective or pathogenic properties of HLA-C in HIV-1 infection.

Such studies might help clarifying the apparent contradiction of the association of higher surface expression of an inhibitory molecule with control of HIV-1 and open new perspectives on the role of HLA-C in the modulation of host-virus interactions.

## Abbreviations

APC: Antigen presenting cell; β2m: β2-microglobulin; CTL: Cytotoxic T lymphocyte; ER: Endoplasmic reticulum; GWAS: Genome wide association study; KIR: Killer cell Ig-like receptor; LILR: Leukocyte immunoglobulin receptor; MHC: Major histocompatibility receptor; NK: Natural killer; SNP: Single nucleotide polymorphism; TCR: T cell receptor.

## Competing interests

The authors declare that they have no competing interests.

## Authors’ contributions

DZ and AB equally contributed in drafting, writing and revising the manuscript. All authors read and approved the final manuscript.

## References

[B1] Davidson WF Kress M Khoury G Jay G Comparison of HLA class I gene sequences. Derivation of locus-specific oligonucleotide probes specific for HLA-A, HLA-B, and HLA-C genes J Biol Chem 1985 260 13414 13423 3863816

[B2] Groothuis TA Griekspoor AC Neijssen JJ Herberts CA Neefjes JJ MHC class I alleles and their exploration of the antigen-processing machinery Immunol Rev 2005 207 60 76 10.1111/j.0105-2896.2005.00305.x 16181327

[B3] Antoniou AN Powis SJ Elliott T Assembly and export of MHC class I peptide ligands Curr Opin Immunol 2003 15 75 81 10.1016/S0952-7915(02)00010-9 12495737

[B4] Cresswell P Ackerman AL Giodini A Peaper DR Wearsch PA Mechanisms of MHC class I-restricted antigen processing and cross-presentation Immunol Rev 2005 207 145 157 10.1111/j.0105-2896.2005.00316.x 16181333

[B5] Vivier E Anfossi N Inhibitory NK-cell receptors on T cells: witness of the past, actors of the future Nat Rev Immunol 2004 4 190 198 10.1038/nri1306 15039756

[B6] Kaiser BK Pizarro JC Kerns J Strong RK Structural basis for NKG2A/CD94 recognition of HLA-E Proc Natl Acad Sci U S A 2008 105 6696 6701 10.1073/pnas.0802736105 18448674 PMC2373352

[B7] Snary D Barnstable CJ Bodmer WF Crumpton MJ Molecular structure of human histocompatibility antigens: the HLA-C series Eur J Immunol 1977 7 580 585 10.1002/eji.1830070816 332508

[B8] Neefjes JJ Ploegh HL Allele and locus-specific differences in cell surface expression and the association of HLA class I heavy chain with beta 2-microglobulin: differential effects of inhibition of glycosylation on class I subunit association Eur J Immunol 1988 18 801 810 10.1002/eji.1830180522 2967765

[B9] Sibilio L Martayan A Setini A Lo Monaco E Tremante E Butler RH Giacomini P A single bottleneck in HLA-C assembly J Biol Chem 2008 283 1267 1274 17956861 10.1074/jbc.M708068200

[B10] Zemmour J Parham P Distinctive polymorphism at the HLA-C locus: implications for the expression of HLA-C J Exp Med 1992 176 937 950 10.1084/jem.176.4.937 1383381 PMC2119399

[B11] Setini A Beretta A De Santis C Meneveri R Martayan A Mazzilli MC Appella E Siccardi AG Natali PG Giacomini P Distinctive features of the alpha 1-domain alpha helix of HLA-C heavy chains free of beta 2-microglobulin Hum Immunol 1996 46 69 81 10.1016/0198-8859(96)00011-0 8727205

[B12] Giacomini P Beretta A Nicotra MR Ciccarelli G Martayan A Cerboni C Lopalco L Bini D Delfino L Ferrara GB HLA-C heavy chains free of beta2-microglobulin: distribution in normal tissues and neoplastic lesions of non-lymphoid origin and interferon-gamma responsiveness Tissue Antigens 1997 50 555 566 10.1111/j.1399-0039.1997.tb02913.x 9458108

[B13] King A Burrows TD Hiby SE Bowen JM Joseph S Verma S Lim PB Gardner L Le Bouteiller P Ziegler A Surface expression of HLA-C antigen by human extravillous trophoblast Placenta 2000 21 376 387 10.1053/plac.1999.0496 10833373

[B14] Schaefer MR Williams M Kulpa DA Blakely PK Yaffee AQ Collins KL A novel trafficking signal within the HLA-C cytoplasmic tail allows regulated expression upon differentiation of macrophages J Immunol 2008 180 7804 7817 18523244 10.4049/jimmunol.180.12.7804 PMC2440697

[B15] Falk CS Schendel DJ HLA-C revisited Ten years of change. Immunol Res 1997 16 203 214 10.1007/BF02786363 9212365

[B16] Colonna M Borsellino G Falco M Ferrara GB Strominger JL HLA-C is the inhibitory ligand that determines dominant resistance to lysis by NK1- and NK2-specific natural killer cells Proc Natl Acad Sci U S A 1993 90 12000 12004 10.1073/pnas.90.24.12000 8265660 PMC48113

[B17] Khakoo SI Thio CL Martin MP Brooks CR Gao X Astemborski J Cheng J Goedert JJ Vlahov D Hilgartner M HLA and NK cell inhibitory receptor genes in resolving hepatitis C virus infection Science 2004 305 872 874 10.1126/science.1097670 15297676

[B18] Rauch A Laird R McKinnon E Telenti A Furrer H Weber R Smillie D Gaudieri S Influence of inhibitory killer immunoglobulin-like receptors and their HLA-C ligands on resolving hepatitis C virus infection Tissue Antigens 2007 69 Suppl 1 237 240 17445209 10.1111/j.1399-0039.2006.773_4.x

[B19] Alter G Martin MP Teigen N Carr WH Suscovich TJ Schneidewind A Streeck H Waring M Meier A Brander C Differential natural killer cell-mediated inhibition of HIV-1 replication based on distinct KIR/HLA subtypes J Exp Med 2007 204 3027 3036 10.1084/jem.20070695 18025129 PMC2118524

[B20] Jennes W Verheyden S Demanet C Adje-Toure CA Vuylsteke B Nkengasong JN Kestens L Cutting edge: resistance to HIV-1 infection among African female sex workers is associated with inhibitory KIR in the absence of their HLA ligands J Immunol 2006 177 6588 6592 17082569 10.4049/jimmunol.177.10.6588

[B21] Martin MP Gao X Lee JH Nelson GW Detels R Goedert JJ Buchbinder S Hoots K Vlahov D Trowsdale J Epistatic interaction between KIR3DS1 and HLA-B delays the progression to AIDS Nat Genet 2002 31 429 434 12134147 10.1038/ng934

[B22] Merino A Malhotra R Morton M Mulenga J Allen S Hunter E Tang J Kaslow RA Impact of a Functional KIR2DS4 Allele on Heterosexual HIV-1 Transmission among Discordant Zambian Couples J Infect Dis 2011 203 487 495 10.1093/infdis/jiq075 21216870 PMC3071217

[B23] Pelak K Need AC Fellay J Shianna KV Feng S Urban TJ Ge D De Luca A Martinez-Picado J Wolinsky SM Copy Number Variation of KIR Genes Influences HIV-1 Control PLoS Biol 2011 9 e1001208 10.1371/journal.pbio.1001208 22140359 PMC3226550

[B24] Tiemessen CT Paximadis M Minevich G Winchester R Shalekoff S Gray GE Sherman GG Coovadia AH Kuhn L Natural killer cell responses to HIV-1 peptides are associated with more activating KIR genes and HLA-C genes of the C1 allotype J Acquir Immune Defic Syndr 2011 57 181 189 10.1097/QAI.0b013e3182174a76 21407082 PMC3280081

[B25] Le Gall S Erdtmann L Benichou S Berlioz-Torrent C Liu L Benarous R Heard JM Schwartz O Nef interacts with the mu subunit of clathrin adaptor complexes and reveals a cryptic sorting signal in MHC I molecules Immunity 1998 8 483 495 10.1016/S1074-7613(00)80553-1 9586638

[B26] Le Gall S Prevost MC Heard JM Schwartz O Human immunodeficiency virus type I Nef independently affects virion incorporation of major histocompatibility complex class I molecules and virus infectivity Virology 1997 229 295 301 10.1006/viro.1996.8417 9123874

[B27] Williams M Roeth JF Kasper MR Fleis RI Przybycin CG Collins KL Direct binding of human immunodeficiency virus type 1 Nef to the major histocompatibility complex class I (MHC-I) cytoplasmic tail disrupts MHC-I trafficking J Virol 2002 76 12173 12184 10.1128/JVI.76.23.12173-12184.2002 12414957 PMC136906

[B28] Schaefer MR Wonderlich ER Roeth JF Leonard JA Collins KL HIV-1 Nef targets MHC-I and CD4 for degradation via a final common beta-COP-dependent pathway in T cells PLoS Pathog 2008 4 e1000131 10.1371/journal.ppat.1000131 18725938 PMC2515349

[B29] Leonard JA Filzen T Carter CC Schaefer M Collins KL HIV-1 Nef disrupts intracellular trafficking of MHC-I, CD4, CD8, and CD28 by distinct pathways that share common elements J Virol 2011 85 6867 6881 10.1128/JVI.00229-11 21543478 PMC3126561

[B30] Cohen GB Gandhi RT Davis DM Mandelboim O Chen BK Strominger JL Baltimore D The selective downregulation of class I major histocompatibility complex proteins by HIV-1 protects HIV-infected cells from NK cells Immunity 1999 10 661 671 10.1016/S1074-7613(00)80065-5 10403641

[B31] Collins KL Chen BK Kalams SA Walker BD Baltimore D HIV-1 Nef protein protects infected primary cells against killing by cytotoxic T lymphocytes Nature 1998 391 397 401 10.1038/34929 9450757

[B32] Kulkarni S Savan R Qi Y Gao X Yuki Y Bass SE Martin MP Hunt P Deeks SG Telenti A Differential microRNA regulation of HLA-C expression and its association with HIV control Nature 2011 472 495 498 10.1038/nature09914 21499264 PMC3084326

[B33] Goulder PJ Edwards A Phillips RE McMichael AJ Identification of a novel HLA-A24-restricted cytotoxic T-lymphocyte epitope within HIV-1 Nef AIDS 1997 11 1883 1884 10.1097/00002030-199715000-00015 9412709

[B34] Jeannet M Sztajzel R Carpentier N Hirschel B Tiercy JM HLA antigens are risk factors for development of AIDS J Acquir Immune Defic Syndr 1989 2 28 32 2783969

[B35] Mann DL Murray C Yarchoan R Blattner WA Goedert JJ HLA antigen frequencies in HIV-1 seropositive disease-free individuals and patients with AIDS J Acquir Immune Defic Syndr 1988 1 13 17 3216293

[B36] Rohowsky-Kochan C Skurnick J Molinaro D Louria D HLA antigens associated with susceptibility/resistance to HIV-1 infection Hum Immunol 1998 59 802 815 10.1016/S0198-8859(98)00086-X 9831136

[B37] Carrington M Nelson GW Martin MP Kissner T Vlahov D Goedert JJ Kaslow R Buchbinder S Hoots K O’ Brien SJ HLA and HIV-1: heterozygote advantage and B*35-Cw*04 disadvantage Science 1999 283 1748 1752 10.1126/science.283.5408.1748 10073943

[B38] Gao X Nelson GW Karacki P Martin MP Phair J Kaslow R Goedert JJ Buchbinder S Hoots K Vlahov D Effect of a single amino acid change in MHC class I molecules on the rate of progression to AIDS N Engl J Med 2001 344 1668 1675 10.1056/NEJM200105313442203 11386265

[B39] Altfeld M Addo MM Rosenberg ES Hecht FM Lee PK Vogel M Yu XG Draenert R Johnston MN Strick D Influence of HLA-B57 on clinical presentation and viral control during acute HIV-1 infection AIDS 2003 17 2581 2591 10.1097/00002030-200312050-00005 14685052

[B40] Costello C Tang J Rivers C Karita E Meizen-Derr J Allen S Kaslow RA HLA-B*5703 independently associated with slower HIV-1 disease progression in Rwandan women AIDS 1999 13 1990 1991 10.1097/00002030-199910010-00031 10513667

[B41] Goulder PJ Phillips RE Colbert RA McAdam S Ogg G Nowak MA Giangrande P Luzzi G Morgan B Edwards A Late escape from an immunodominant cytotoxic T-lymphocyte response associated with progression to AIDS Nat Med 1997 3 212 217 10.1038/nm0297-212 9018241

[B42] Goulder PJ Watkins DI Impact of MHC class I diversity on immune control of immunodeficiency virus replication Nat Rev Immunol 2008 8 619 630 10.1038/nri2357 18617886 PMC2963026

[B43] Kiepiela P Leslie AJ Honeyborne I Ramduth D Thobakgale C Chetty S Rathnavalu P Moore C Pfafferott KJ Hilton L Dominant influence of HLA-B in mediating the potential co-evolution of HIV and HLA Nature 2004 432 769 775 10.1038/nature03113 15592417

[B44] Lazaryan A Lobashevsky E Mulenga J Karita E Allen S Tang J Kaslow RA Human leukocyte antigen B58 supertype and human immunodeficiency virus type 1 infection in native Africans J Virol 2006 80 6056 6060 10.1128/JVI.02119-05 16731944 PMC1472610

[B45] Leslie A Matthews PC Listgarten J Carlson JM Kadie C Ndung’u T Brander C Coovadia H Walker BD Heckerman D Goulder PJ Additive contribution of HLA class I alleles in the immune control of HIV-1 infection J Virol 2010 84 9879 9888 10.1128/JVI.00320-10 20660184 PMC2937780

[B46] Migueles SA Sabbaghian MS Shupert WL Bettinotti MP Marincola FM Martino L Hallahan CW Selig SM Schwartz D Sullivan J Connors M HLA B*5701 is highly associated with restriction of virus replication in a subgroup of HIV-infected long term nonprogressors Proc Natl Acad Sci U S A 2000 97 2709 2714 10.1073/pnas.050567397 10694578 PMC15994

[B47] O’ Brien SJ Gao X Carrington M HLA and AIDS: a cautionary tale Trends Mol Med 2001 7 379 381 10.1016/S1471-4914(01)02131-1 11530315

[B48] Salgado M Simon A Sanz-Minguela B Rallon NI Lopez M Vicario JL Benito JM Rodes B An Additive Effect of Protective Host Genetic Factors Correlates With HIV Nonprogression Status J Acquir Immune Defic Syndr 2011 56 300 305 10.1097/QAI.0b013e3182036f14 21084992

[B49] Fitzgerald DW Janes H Robertson M Coombs R Frank I Gilbert P Loufty M Mehrotra D Duerr A An Ad5-Vectored HIV-1 Vaccine Elicits Cell-mediated Immunity but does not Affect Disease Progression in HIV-1-infected Male Subjects: Results From a Randomized Placebo-Controlled Trial (The Step Study) J Infect Dis 2011 203 765 772 10.1093/infdis/jiq114 21343146 PMC3119328

[B50] Migueles SA Rood JE Berkley AM Guo T Mendoza D Patamawenu A Hallahan CW Cogliano NA Frahm N Duerr A Trivalent Adenovirus Type 5 HIV Recombinant Vaccine Primes for Modest Cytotoxic Capacity That Is Greatest in Humans with Protective HLA Class I Alleles PLoS Pathog 2011 7 e1002002 10.1371/journal.ppat.1002002 21383976 PMC3044701

[B51] Dong T Zhang Y Xu KY Yan H James I Peng Y Blais ME Gaudieri S Chen X Lun W Extensive HLA-driven viral diversity following a narrow-source HIV-1 outbreak in rural China Blood 2011 118 98 106 10.1182/blood-2010-06-291963 21562042 PMC3743462

[B52] McMichael AJ Jones EY Genetics. First-class control of HIV-1 Science 2010 330 1488 1490 10.1126/science.1200035 21148380

[B53] Singh KK Gray PK Wang Y Fenton T Trout R Spector SA HLA Alleles are Associated with Altered Risk for Disease Progression and Central Nervous System Impairment of HIV-Infected Children J Acquir Immune Defic Syndr 2011 57 32 39 10.1097/QAI.0b013e3182119244 21283014 PMC3107908

[B54] Honda K Zheng N Murakoshi H Hashimoto M Sakai K Borghan MA Chikata T Koyanagi M Tamura Y Gatanaga H Selection of escape mutant by HLA-C-restricted HIV-1 Pol-specific cytotoxic T lymphocytes carrying strong ability to suppress HIV-1 replication Eur J Immunol 2011 41 97 106 10.1002/eji.201040841 21182081

[B55] Carrington M Martin MP van Bergen J KIR-HLA intercourse in HIV disease Trends Microbiol 2008 16 620 627 10.1016/j.tim.2008.09.002 18976921 PMC3463869

[B56] Fellay J Shianna KV Telenti A Goldstein DB Host genetics and HIV-1: the final phase? PLoS Pathog 2010 6 e1001033 10.1371/journal.ppat.1001033 20976252 PMC2954832

[B57] Fellay J Shianna KV Ge D Colombo S Ledergerber B Weale M Zhang K Gumbs C Castagna A Cossarizza A A whole-genome association study of major determinants for host control of HIV-1 Science 2007 317 944 947 10.1126/science.1143767 17641165 PMC1991296

[B58] de Bakker PI McVean G Sabeti PC Miretti MM Green T Marchini J Ke X Monsuur AJ Whittaker P Delgado M A high-resolution HLA and SNP haplotype map for disease association studies in the extended human MHC Nat Genet 2006 38 1166 1172 10.1038/ng1885 16998491 PMC2670196

[B59] Study TIHC The major genetic determinants of HIV-1 control affect HLA class I peptide presentation Science 2010 330 1551 1557 21051598 10.1126/science.1195271 PMC3235490

[B60] Corrah TW Goonetilleke N Kopycinski J Deeks SG Cohen MS Borrow P McMichael A Brackenridge S Reappraisal of the relationship between the HIV-1-protective single-nucleotide polymorphism 35 kilobases upstream of the HLA-C gene and surface HLA-C expression J Virol 2011 85 3367 3374 10.1128/JVI.02276-10 21248048 PMC3067890

[B61] Specht A Telenti A Martinez R Fellay J Bailes E Evans DT Carrington M Hahn BH Goldstein DB Kirchhoff F Counteraction of HLA-C-mediated immune control of HIV-1 by Nef J Virol 2010 84 7300 7311 10.1128/JVI.00619-10 20463068 PMC2898263

[B62] O’ Huigin C Kulkarni S Xu Y Deng Z Kidd J Kidd K Gao X Carrington M The Molecular Origin and Consequences of Escape from miRNA Regulation by HLA-C Alleles Am J Hum Genet 2011 89 424 431 10.1016/j.ajhg.2011.07.024 21907013 PMC3169826

[B63] Adnan S Balamurugan A Trocha A Bennett MS Ng HL Ali A Brander C Yang OO Nef interference with HIV-1-specific CTL antiviral activity is epitope specific Blood 2006 108 3414 3419 10.1182/blood-2006-06-030668 16882705 PMC1895430

[B64] Buseyne F Stevanovic S Rammensee HG Riviere Y Characterization of an HIV-1 p24gag epitope recognized by a CD8+ cytotoxic T-cell clone Immunol Lett 1997 55 145 149 10.1016/S0165-2478(97)02696-5 9161880

[B65] Johnson RP Trocha A Buchanan TM Walker BD Recognition of a highly conserved region of human immunodeficiency virus type 1 gp120 by an HLA-Cw4-restricted cytotoxic T-lymphocyte clone J Virol 1993 67 438 445 7677956 10.1128/jvi.67.1.438-445.1993 PMC237380

[B66] Littaua RA Oldstone MB Takeda A Debouck C Wong JT Tuazon CU Moss B Kievits F Ennis FA An HLA-C-restricted CD8+ cytotoxic T-lymphocyte clone recognizes a highly conserved epitope on human immunodeficiency virus type 1 gag J Virol 1991 65 4051 4056 1712857 10.1128/jvi.65.8.4051-4056.1991 PMC248836

[B67] Makadzange AT Gillespie G Dong T Kiama P Bwayo J Kimani J Plummer F Easterbrook P Rowland-Jones SL Characterization of an HLA-C-restricted CTL response in chronic HIV infection Eur J Immunol 2010 40 1036 1041 10.1002/eji.200939634 20104487

[B68] Rousseau CM Daniels MG Carlson JM Kadie C Crawford H Prendergast A Matthews P Payne R Rolland M Raugi DN HLA class I-driven evolution of human immunodeficiency virus type 1 subtype c proteome: immune escape and viral load J Virol 2008 82 6434 6446 10.1128/JVI.02455-07 18434400 PMC2447109

[B69] Buranapraditkun S Hempel U Pitakpolrat P Allgaier RL Thantivorasit P Lorenzen SI Sirivichayakul S Hildebrand WH Altfeld M Brander C A Novel Immunodominant CD8+ T Cell Response Restricted by a Common HLA-C Allele Targets a Conserved Region of Gag HIV-1 Clade CRF01_AE Infected Thais PLoS One 2011 6 e23603 10.1371/journal.pone.0023603 21887282 PMC3161737

[B70] Nehete PN Lewis DE Tang DN Pollack MS Sastry KJ Presence of HLA-C-restricted cytotoxic T-lymphocyte responses in long-term nonprogressors infected with human immunodeficiency virus Viral Immunol 1998 11 119 129 10.1089/vim.1998.11.119 9918403

[B71] Wagtmann N Rajagopalan S Winter CC Peruzzi M Long EO Killer cell inhibitory receptors specific for HLA-C and HLA-B identified by direct binding and by functional transfer Immunity 1995 3 801 809 10.1016/1074-7613(95)90069-1 8777725

[B72] Colonna M Samaridis J Cloning of immunoglobulin-superfamily members associated with HLA-C and HLA-B recognition by human natural killer cells Science 1995 268 405 408 10.1126/science.7716543 7716543

[B73] Carter AM Comparative studies of placentation and immunology in non-human primates suggest a scenario for the evolution of deep trophoblast invasion and an explanation for human pregnancy disorders Reproduction 2011 141 391 396 10.1530/REP-10-0530 21273370

[B74] Biassoni R Cantoni C Falco M Verdiani S Bottino C Vitale M Conte R Poggi A Moretta A Moretta L The human leukocyte antigen (HLA)-C-specific “activatory” or “inhibitory” natural killer cell receptors display highly homologous extracellular domains but differ in their transmembrane and intracytoplasmic portions J Exp Med 1996 183 645 650 10.1084/jem.183.2.645 8627176 PMC2192451

[B75] Biassoni R Falco M Cambiaggi A Costa P Verdiani S Pende D Conte R Di Donato C Parham P Moretta L Amino acid substitutions can influence the natural killer (NK)-mediated recognition of HLA-C molecules. Role of serine-77 and lysine-80 in the target cell protection from lysis mediated by “group 2” or “group 1” NK clones J Exp Med 1995 182 605 609 10.1084/jem.182.2.605 7629517 PMC2192139

[B76] Moretta A Vitale M Bottino C Orengo AM Morelli L Augugliaro R Barbaresi M Ciccone E Moretta L P58 molecules as putative receptors for major histocompatibility complex (MHC) class I molecules in human natural killer (NK) cells. Anti-p58 antibodies reconstitute lysis of MHC class I-protected cells in NK clones displaying different specificities J Exp Med 1993 178 597 604 10.1084/jem.178.2.597 8340759 PMC2191136

[B77] Alter G Heckerman D Schneidewind A Fadda L Kadie CM Carlson JM Oniangue-Ndza C Martin M Li B Khakoo SI HIV-1 adaptation to NK-cell-mediated immune pressure Nature 2011 476 96 100 10.1038/nature10237 21814282 PMC3194000

[B78] van Bergen J Koning F The tortoise and the hare: slowly evolving T-cell responses take hastily evolving KIR Immunology 2010 131 301 309 10.1111/j.1365-2567.2010.03337.x 20722764 PMC2996550

[B79] Alter G Rihn S Streeck H Teigen N Piechocka-Trocha A Moss K Cohen K Meier A Pereyra F Walker B Altfeld M Ligand-independent exhaustion of killer immunoglobulin-like receptor-positive CD8+ T cells in human immunodeficiency virus type 1 infection J Virol 2008 82 9668 9677 10.1128/JVI.00341-08 18579582 PMC2546972

[B80] Esser MT Graham DR Coren LV Trubey CM Bess JW Arthur LO Ott DE Lifson JD Differential incorporation of CD45, CD80 (B7-1), CD86 (B7-2), and major histocompatibility complex class I and II molecules into human immunodeficiency virus type 1 virions and microvesicles: implications for viral pathogenesis and immune regulation J Virol 2001 75 6173 6182 10.1128/JVI.75.13.6173-6182.2001 11390619 PMC114333

[B81] Hunsmann G Dormont D Le Grand R Cranage M Greenaway P Stahl-Hennig C Rossi G Verani P Stott J Kitchin P Protection of macaques against simian immunodeficiency virus infection with inactivated vaccines: comparison of adjuvants, doses and challenge viruses. The European Concerted Action on’ Macaque Models for AIDS Research’ Vaccine 1995 13 295 300 7631516

[B82] Ott DE Cellular proteins in HIV virions Rev Med Virol 1997 7 167 180 10.1002/(SICI)1099-1654(199709)7:3<167::AID-RMV199>3.0.CO;2-K 10398481

[B83] Putkonen P Thorstensson R Cranage M Nilsson C Ghavamzadeh L Albert J Greenaway P Biberfeld G A formalin inactivated whole SIVmac vaccine in Ribi adjuvant protects against homologous and heterologous SIV challenge J Med Primatol 1992 21 108 112 1433260

[B84] Stott EJ Anti-cell antibody in macaques Nature 1991 353 393 1815549 10.1038/353393a0

[B85] Tremblay MJ Fortin JF Cantin R The acquisition of host-encoded proteins by nascent HIV-1 Immunol Today 1998 19 346 351 10.1016/S0167-5699(98)01286-9 9709501

[B86] Lakhashe SK Thakar MR Bharucha KE Paranjape RS Quantitation of HLA proteins incorporated by human immunodeficiency virus type 1 and assessment of neutralizing activity of anti-HLA antibodies J Virol 2008 82 428 434 10.1128/JVI.00638-07 17942547 PMC2224394

[B87] Arthur LO Bess JW Sowder RC Benveniste RE Mann DL Chermann JC Henderson LE Cellular proteins bound to immunodeficiency viruses: implications for pathogenesis and vaccines Science 1992 258 1935 1938 10.1126/science.1470916 1470916

[B88] Zhu P Liu J Bess J Chertova E Lifson JD Grise H Ofek GA Taylor KA Roux KH Distribution and three-dimensional structure of AIDS virus envelope spikes Nature 2006 441 847 852 10.1038/nature04817 16728975

[B89] Cantin R Fortin JF Lamontagne G Tremblay M The presence of host-derived HLA-DR1 on human immunodeficiency virus type 1 increases viral infectivity J Virol 1997 71 1922 1930 9032323 10.1128/jvi.71.3.1922-1930.1997 PMC191270

[B90] Cantin R Fortin JF Lamontagne G Tremblay M The acquisition of host-derived major histocompatibility complex class II glycoproteins by human immunodeficiency virus type 1 accelerates the process of virus entry and infection in human T-lymphoid cells Blood 1997 90 1091 1100 9242540

[B91] de Santis C Robbioni P Longhi R Carrow E Siccardi AG Beretta A Role of HLA class I in HIV type 1-induced syncytium formation AIDS Res Hum Retroviruses 1996 12 1031 1040 10.1089/aid.1996.12.1031 8827219

[B92] Cosma A Blanc D Braun J Quillent C Barassi C Moog C Klasen S Spire B Scarlatti G Pesenti E Enhanced HIV infectivity and changes in GP120 conformation associated with viral incorporation of human leucocyte antigen class I molecules AIDS 1999 13 2033 2042 10.1097/00002030-199910220-00005 10546855

[B93] Matucci A Rossolillo P Baroni M Siccardi AG Beretta A Zipeto D HLA-C increases HIV-1 infectivity and is associated with gp120 Retrovirology 2008 5 68 10.1186/1742-4690-5-68 18673537 PMC2531131

[B94] Beretta A Grassi F Pelagi M Clivio A Parravicini C Giovinazzo G Andronico F Lopalco L Verani P Butto S HIV env glycoprotein shares a cross-reacting epitope with a surface protein present on activated human monocytes and involved in antigen presentation Eur J Immunol 1987 17 1793 1798 10.1002/eji.1830171218 2446880

[B95] Grassi F Meneveri R Gullberg M Lopalco L Rossi GB Lanza P De Santis C Brattsand G Butto S Ginelli E Human immunodeficiency virus type 1 gp120 mimics a hidden monomorphic epitope borne by class I major histocompatibility complex heavy chains J Exp Med 1991 174 53 62 10.1084/jem.174.1.53 1711567 PMC2118897

[B96] Fan QR Wiley DC Structure of human histocompatibility leukocyte antigen (HLA)-Cw4, a ligand for the KIR2D natural killer cell inhibitory receptor J Exp Med 1999 190 113 123 10.1084/jem.190.1.113 10429675 PMC2195553

[B97] Finzi A Brunet A Xiao Y Thibodeau J Cohen EA Major histocompatibility complex class II molecules promote human immunodeficiency virus type 1 assembly and budding to late endosomal/multivesicular body compartments J Virol 2006 80 9789 9797 10.1128/JVI.01055-06 16973583 PMC1617259

[B98] Gottlinger HG The HIV-1 assembly machine AIDS 2001 15 Suppl 5 S13 S20 11816161 10.1097/00002030-200100005-00003

[B99] MacAry PA Lindsay M Scott MA Craig JI Luzio JP Lehner PJ Mobilization of MHC class I molecules from late endosomes to the cell surface following activation of CD34-derived human Langerhans cells Proc Natl Acad Sci U S A 2001 98 3982 3987 10.1073/pnas.071477498 11274420 PMC31165

[B100] Arosa FA Santos SG Powis SJ Open conformers: the hidden face of MHC-I molecules Trends Immunol 2007 28 115 123 10.1016/j.it.2007.01.002 17261379

[B101] Martayan A Fraioli R Giorda E Setini A Ciccarelli G Delfino L Ferrara GB Giacomini P Biosynthesis of HLA-C heavy chains in melanoma cells with multiple defects in the expression of HLA-A, -B, -C molecules Br J Cancer 1999 80 639 649 10.1038/sj.bjc.6690405 10360639 PMC2362293

[B102] Mothe B Llano A Ibarrondo J Zamarreno J Schiaulini M Miranda C Ruiz-Riol M Berger CT Herrero MJ Palou E CTL Responses of High Functional Avidity and Broad Variant Cross-Reactivity Are Associated with HIV Control PLoS One 2012 7 e29717 10.1371/journal.pone.0029717 22238642 PMC3251596

